# History of the Late J. Kearney Rodgers, M. D.

**Published:** 1852-02

**Authors:** Alexander E. Hosack

**Affiliations:** New York


ART. III.—*History of the Case of the late John Kearney Rodgers, M. D.* By ALEXANDER E. HOSACK, M. D. of New York.*

INTRODUCTION.—The late Dr. J. KEARNEY RODGERS died, Sunday, the 9th day of November, 1851, of pyaëmia, consequent upon congestion of the liver.

However much we have to deplore the loss of so eminent and distinguished a surgeon, we have the more to regret that there should have been a difference of opinion entertained by his attending physicians, as regards the diagnosis and treatment of the disease which ended so fatally.

Nearly allied to the deceased as I am, it is with the greatest reluctance I have ventured upon the task of publishing a history of the case, which I shall do as correctly as possible; narrating all the circumstances connected with the differences to which I have alluded. This I am constrained to do, in consequence of the numerous misapprehensions that have been suffered to go abroad calculated to disparage me with my fellow-practitioners. The medical public will, after being possessed of all the facts,

---

*From a pamphlet sold by C. S. Francis & Co., Stringer & Townsend, &c., New York, 1851.

be enabled to determine, whether I was justified in the opinion early entertained and expressed, (and from which I never for a moment swerved,) as to the true cause and correct diagnosis of the disease which terminated his life; and whether I was correct in the course I pursued in withdrawing from the consultation at a time of so much interest and responsibility.

*History.*—Dr. Rodgers, although naturally of a good constitution, has once in his life suffered from a severe attack of illness, which occurred about sixteen years ago, during the winter of 1835. He became extremely attenuated, and serious doubts were entertained of his perfect recovery; his disease was never satisfactorily understood. His friends, however, regarding it as dyspepsia, advised a voyage to the West Indies, from whence he returned greatly improved, and soon after was perfectly restored to health. Since then he has been apparently well until the commencement of the present year, when he has occasionally been annoyed by looseness of the bowels, with a slight feeling of discomfort in the abdomen, and only on two different occasions has he been obliged to confine himself to his chamber, and then but for a day or two at a time. He, regarding it as an inconvenience only, resorted occasionally to a few drops of laudanum during the day, or a glass of brandy and water at his dinner, which exerted a controlling influence over it. The first of these attacks occurred in the month of July, and the second in August, while enjoying himself for a few weeks at Long Branch on the seashore.

This, then, embraces all that appertains to his ill health up to the 9th of October, when he was seized with the illness of which he died. On the evening of that day, after returning from a visit to a patient, he complained of not feeling well; he retired at his usual hour, and was awakened about midnight by a sense of coldness, and had a slight chill; complaining of nausea, he called for a basin, and threw off, as he informed me, about two mouthfuls of pure bile; he refused to take warm water to rinse his stomach, as was urged upon him, and soon after fell asleep. He rose at the usual hour in the morning, saying he was better, though complaining of uneasiness in the right side, and slight pain in the bowels. He partook of breakfast as usual, and visited such patients as were necessary to be seen during the day.

On Saturday, the 11th instant, Dr. Dubois saw him for the first time, and suggested a seidlitz powder, which was taken. On Sunday morning, Dr. Wilkes stopped in on a visit to the family, when Dr. Rodgers consulted him about his symptoms, which Dr. Wilkes regarded as functional disorder of the liver, and accordingly advised the use of blue mass, and actually procured the pills for him, urging him to take two immediately, to be followed by a seidlitz draught. At Dr. Rodgers's request, Dr. Wilkes called at my house that morning to inform me of the Doctor's illness, and of his desire to see me; not receiving him the message until too late in the evening, I called upon the following morning, Oct. 13th. I found him free from fever, with a white, slightly coated tongue. Although he complained of pain in the bowels and of a slight uneasiness in the hypochondriac region, for which he had applied a mustard cataplasm, he did not wince upon pressure.—I saw him the next day, Tuesday, the 14th, and found him much the same, with the exception of a slightly accelerated pulse, full and compressible, with a tongue assuming a dingy hue at its base.

Regarding these symptoms as indicative of a biliary congestion, which opinion I stated to him, I advised him to take an emetic or ten grains of calomel; but he, having a particular dislike to both these remedies, declined. I was then informed by him that he had been exposed to miasma by attending on a case of bilious remittent fever at Flatbush, Long Island, and he remarked that he might possibly have there contracted the disease. I told him, in reply, that it was quite possible it might be the incipient stage of bilious remittent fever, and questioned him as to the frequency of his visits, and the hour of the day he had been exposed to the effluvium of that district of country; his reply was, "never after four o'clock in the afternoon."

I then said that it wanted the phenomena of fever, the excessive heat, and dryness of skin, the constricted pulse, &c. Taking into consideration the former symptoms, his vomiting bile, dingy skin, and his then present appearance, I still adhered to the opinion I had first expressed of the liver being the seat of the disease. On the evening of this day at Dr. Rodgers's request, I consulted with Dr. Dubois, to whom I also expressed the same opinion, and suggested the use of calomel. Dr. Dubois had already formed and expressed the opinion of the disease being that of bilious remittent fever, from which opinion he never changed.

On Wednesday, the 15th, we again met in consultation; he had had a restless night, and upon examination he was discovered to be quite jaundiced; tongue and pulse about the same as on the preceding day. The patient called our attention to the yellowness of the skin as manifested on the hands and lower extremities, which, on close inspection, proved to be decided Icterus. I immediately remarked to him, in presence of Dr. Dubois, that the tale was now told, and that he, the patient, must immediately commence on calomel.

It is to be remembered that at this time he did not wince, or manifest the least pain any where, upon being kneaded, or upon turning in bed, rising, or lying down.—As he objected to calomel, fearing salivation, I could only succeed in persuading him to take the common Icteric Pill (a prescription well known to those who attended my father's Lectures on the Practice of Physic), to which my colleague consented. It contained calomel which was not likely to salivate, by being in combination with other medicines.

He commenced taking it in the evening, and continued it all the day following: it operated gently. Friday morning, the 17th, he had slept well, and expressed himself as feeling better, and said that the day previous was the best day he had had. His condition was much the same throughout the day, except that sallowness, which was more manifest.

Dr. Delafield joined us in consultation on Friday evening, Oct. 17th, to whom I said that I presumed that Dr. Dubois had informed him of the previous history of the case; to which he assented. I then stated to Dr. Delafield my views of the case, and the prescription I had prevailed upon Dr. R. to take, and which he was permitted to continue without any intimation to me from Dr. Delafield of different views entertained by himself as regarded the disease.

I did not attend at the appointed hour on the following morning, Saturday the 18th, in consequence of some professional engagements. In the evening, at the hour appointed, I was informed that the remedies suggested by myself (as above alluded to) had been discontinued at the morning consultation, my associates regarding his disease as bilious remittent fever, and it was so expressed by them. In this I decidedly differed in opinion, as well as in the remedies resorted to, which were the usual febrifuge medicines.

His symptoms at this time were general restlessness, imperfect sleep, depression of spirits, anxious countenance, slight fever, increased sallowness, accelerated pulse, and at times moderate perspiration. These symptoms continued much the same from the 19th of October to the 22d, (when rigors first appeared), with the exception of the increased frequency of the pulse, which varied from

95 to 120, but usually at the standard of 110, always full and compressible. The rigors recurred at irregular intervals, sometimes in the night and at any hour during the day; at first, however, there were but one or two in the 24 hours: they soon after increased in frequency: jaundice more apparent.

The rigors were always immediately followed by profuse perspirations—the pulse, as observed by myself, was 110 just before, during, and after each rigor, which was always accompanied by more or less perspiration, and followed directly after by a profuse sweat. The gentlemen in consultation, with the view of opposing the chills, prescribed (in opposition to my judgment) quinine in 10 and 5 grain doses, at intervals of several hours, which was continued for several successive days. The positive effect of quinine was in due time made manifest, by *ringing in the ears* and almost *total deafness;* in consequence of which it was discontinued on the 29th of October. Dr. Delafield remarked in consultation at the same time, that it was very evident that the quinine was doing no good, that there was something in the case that he could not understand: to which I replied, that I understood it, and could account for it in a very satisfactory manner. The aromatic sulphuric acid was also prescribed in large doses, for the purpose of arresting the excessive sweats. The strong tincture of aconite was also administered in doses of one drop each.

At this stage of the disease, finding myself opposed in opinion, and the patient evidently getting worse, in one of my visits to Dr. Rodgers, (when alone with him in the room,) I was prompted by my deep solicitude and anxiety on his account, to urge upon him the necessity of further medical advice. Seated by his bedside, I asked him to permit me to call in Dr. Wilkes (as I daily communicated with him in regard to his illness) who had seen him, and thought as I did. I then said, "If you will permit me to do so, and follow our advice, you will get well; I promise you, you will recover." To which he replied, after a moment's reflection (being still biased with the idea of fever), "If I do, I can never recover, and I have all confidence in Delafield." It is deserving of remark, that a few days after, finding he was growing worse, he said to a member of his family, "There is something in my case that Dr. Delafield does not understand, nor I either."

At this period of his disease, the 23d of October, on the day after the appearance of the rigors, I again remarked to Drs. Delafield and Dubois, in consultation, that it was not bilious remittent fever, as it wanted the characteristics of fever, and that it was the consequence of unremoved obstruction in the liver. Dr. Delafield then asked me where I located the disease; to which I replied, in the liver, either arising from obstruction in the "ductus communis choledochus" by biliary calculi, or from inspissated bile in its *transit from the ramifications of the portal vein in the liver to the biliar ducts*, and that these *chills*, so denominated by my associates, were rigors, indicative of matter forming, if not already formed; to which he replied, "I hope not," and said "I had nothing to stand upon, or to sustain me in my views." I then asked him, Are these symptoms nothing? this jaundice, this condition of the tongue, these rigors, followed by profuse sweats, and this peculiar, rapid, full, compressible pulse, and comparatively moderate heat of skin; are all these symptoms nothing? In answer to which Dr. Delafield remarked that this yellowness and other symptoms, as above observed, were frequently met with in bilious fevers; to which I replied, "I have, then, yet to learn what bilious fever is."

These same remarks were repeated in substance by me to Dr. Swett at the time of my withdrawing from the consultation, on the morning of the 3d of November (he having been called in on the 1st inst.), with the additional observation, that in fever there should be remissions and exacerbations, a furred tongue, and a burning, hot and dry skin.

I then drew a parallel between the pulse of bilious fever and the pulse of matter forming, or just formed, as follows: In bilious fever, during the exacerbations, it would be small, corded, and quick, varying in frequency (from the remitting stage to the highest degree of fever) from an almost natural state to 120, at which height of pulse perspiration would commence; whereas the pulse indicative of matter would be always frequent, full and soft, varying but little from 95 to 110, while the skin most of the time would be comparatively cool. The pulse full and soft is 110 just before, during, and after each rigor, which is immediately followed by profuse perspiration.

After these remarks I observed, "With these existing symptoms, gentlemen, how is it possible you can see things with such different eyes from me?" Placing my hand upon my knee, I said, "It is as plain to me as if matter were formed in this joint which the knife can release; but in this case we have no such opportunity. I have no doubt that matter, if not already formed, will form, and terminate fatally."* I then added, "From my deep solicitude I have scarcely slept for the last two nights, and have tried to reconcile my opinion with yours, but the more I reflect upon the subject, the more I am convinced that I am correct in my views."

I then withdrew from the consultation, remarking that I did so with all respect and deference to them, but that I could not remain to trammel them by my presence,

---

*These remarks were repeated to several of my medical friends, and also to Dr. J. Mason Warren, of Boston, who was on a visit to New York at that time.

or witness a course of treatment I could not sanction.*

From my connection with Dr. Rodgers, I had the privilege of visiting him at all times, during his last illness, and had ample opportunity to observe the progress of his disease and the treatment pursued by the physicians left in sole charge of him. Upon my proposing to withdraw from the consultation it was suggested by Dr. Delafield that the patient might be disturbed by knowing that I had done so: in reply I assured him that I would take care he should not be apprised of it, and would therefore visit him at the accustomed hour of consultation, without retiring afterwards to the council chamber. Under these circumstances I do not feel called upon to offer any excuse for continuing the history of his disease to its termination.

Having last spoken of the disease up to the 28th October, when the rigors became most frequent, and on the 29th inst., when there was a continuance of coldness, and when the rigors succeeded rapidly, one upon the other, (which circumstance I conceive to be indicative of matter formed,)† I will proceed with the details of the case.

On the 29th of October he had a succession of chilly feelings for an hour, which were immediately succeeded by a severe rigor, making in all five during the day. He

---

*Dr. Swett at the first consultation, upon his being called in, (in reply to Dr. Delafield) said "that the patient ought to take three grains of mercury three times a day, and that he should be cupped over the region of the liver as there was congestion of the liver," which was objected to by Dr. Delafield. Dr. Swett, the following day, visited the patient morning and noon alone, and in the evening consultation wholly changed his opinion of the disease, agreeing with the other two gentlemen that it was bilious remittent fever.

† Eisenmann asserts, that where two chills come in immediate succession, it is indicative of the begginning of suppuration; these double rigors may recur during the formation of matter. (See Eisenmann.)

was much depressed in spirits and betrayed great exhaustion of the vital powers. Pulse quick, slightly diminished in calibre and weak; cheeks flushed, as had been the case daily for some time previous. At this period I consider, that matter was already formed. On this day the quinine was discontinued, and tincture of aconite in drop doses was substituted at two hours interval.

On Thursday, Oct. 30, he had a repetition of the chilly feelings, and three rigors of twenty or thirty minutes duration, followed immediately by profuse sweat;—symptoms in other respects much the same. Aconite and effervescent draught continued.

Friday, Oct. 31. Three rigors, followed as usual by perspiration—pulse about the same as on the two preceding days—symptoms in other respects unaltered. A single dose of twenty drops of aromatic sulphuric acid was resorted to with the view to check the excessive perspiration.

Saturday, Nov. 1. Renewal of chilly feelings twice during the twenty-four hours, each succeeded by profuse perspiration—one rigor only during the day, which continued twenty minutes—pulse about the same—countenance expressive of great anxiety. At this stage of the disease, perspiration appeared from time to time without any chilly feelings, or rigors, thereby betraying great exhaustion of the vital energies. Nitro-muriatic acid in the usual proportions was given internally, and was used as a "pede luvium;" it was also applied to the surface of the body and lower extremities. Jaundice was regularly declining, having commenced to do so about the 27th.

Sunday, Nov. 2. Perspiration as usual continued and profuse—tongue as heretofore slightly coated. This day one rigor only, which lasted twenty minutes—much exhaustion. Jaundice rapidly declining; the urine by analysis exhibited a large proportion of bile.

Monday, Nov. 3. But one rigor on this day—much perspiration and coldness of the nose and extremities—other symptoms the same as on the last preceding days. The hectic flush was at intervals still manifest.

Tuesday, Nov. 4. Quinine was again resorted to, but in smaller doses—*two or three grains I believe every two hours—aconite continued, and nitro-muriatic acid given simultaneously with the quinine.

Wednesday, Nov. 5. Awakened by pain in the bowels, or directly over the region of the stomach, as he informed me, which lasted twenty-five minutes, for which linen, saturated with laudanum, was applied. There was slight tumefaction of the abdomen—the pain and general soreness recurred several hours after, for which peppermint was administered; warm flannels were applied to the bowels, but not producing relief, a mustard plaster was substituted. Quinine continued, and on one occasion associated with *a teaspoonful of laudanam* with the view of preventing a recurrence of the rigors! At this time his pulse was small and tremulous, and very rapid, countenance haggard, and great dejection of spirits which continued throughout.

Thursday, Nov. 6. Pulse small, tremulous and quick; perspiration slight, but almost constant, desponding countenance and great depression of spirits, as was evident by different expressions used to those surrounding his bed. Pain in the bowels extending over to the left hypochondriac region. Quinine and nutritious sick room diet, such as beef tea, sago, brandy and water, and chicken broth, were freely resorted to.

Friday, Nov. 7. Having passed a sleepless night, he

---

*For any inaccuracies of dates, or of proportions of medicines not specified, I must plead in excuse my inability to obtain any information from the apothecary, who was instructed *by the attending physicians, to refuse to give me a copy of the prescriptions.*

betrayed great exhaustion—pulse feeble and frequent, countenance dejected, expressive of much anxiety; extremities cold, with much soreness of the abdomen: the same sustaining diet, and stimulants as on the day previous, were continued: fomentations and cataplasms were from time to time applied to the stomach and bowels.

Saturday, Nov. 8. Increased debility, and tenderness of the bowels, as on the preceding day: small, feeble, and quick pulse—great restlessness, evident sinking of the vital powers—hiccup in the morning of an hour and upwards duration, which reappeared in the evening: mind somewhat confused. He continued to decline until three o'clock, A. M., at which time he ceased to breathe.*

As a journal from the 23d of October was kept by the family, I will offer no apology for transcribing it, as it exhibits the bed-room record of the phases of the disease, and remedies resorted to. (Omitted, as containing no new facts.)

*Post-mortem Examination of the Body of Dr. J. Kearney Rodgers.* By C. G. Isaacs, M. D. [I must tender my thanks to Dr. Isaacs for so politely furnishing me with this copy.]

On laying open the cavity of the abdomen, it was found to contain a large quantity of semi-purulent matter, with flakes of coagulable lymph, which adhered to the peritoneal lining of the abdomen and to the serous surface of the intestines. The omentum was partially spread out over the small intestines, and adhered slightly to their surface. On carefully separating with the knife, the mesenteric border of the small intestines from the mesen-

*At the early stage of the disease the bowels were relieved by the ordinary aperient medicine injections, &c. The stools were generally of a dark or brown color: on one occasion, on the 18th or 19th of October, I observed after the effects of an enema, that the dejections were of a light clay-colored appearance.

tery, numerous small drops of purulent matter were observed, on the separated edge of the mesentery, and on the corresponding border of the intestine—along the whole extent of divided surface from the termination of the ilium, up to the commencement of the jejunum. (This purulent matter was afterwards ascertained to have issued from the cut orifices of the mesenteric veins.) On cutting into the mesentery, several small depots of purulent matter were found, containing from half an ounce to an ounce and a half of fluid. These were situated in the cellular tissue, between the laminæ of the mesentery. The large and small intestines were removed and opened; but did not exhibit any appearance of disease (except on their peritoneal surface). The stomach, spleen and pancreas were healthy. The liver was removed, and the biliary ducts carefully traced out and opened, and appeared healthy. The liver was then laid upon its inferior surface, and upon making an incision upon its upper, or convex surface, several small points of purulent matter were perceived. On carefully examining these, the purulent matter was found to issue from the cut orifices of the branches of the vena portæ. Incisions were then made into the substance of the liver, in various places, and the same appearances were observed in the cut portions. The trunk of the vena portæ was now examined, and presented the appearance and feel of a firm, hard cylinder, and when opened, was found to be filled with coagulable lymph and semi-purulent matter, and a few small clots of blood. This vein, upon being traced into the right and left lobes of the liver, exhibited the same appearance in its branches, as far as their third and fourth divisions. Subsequently (next day) a small portion of the liver, placed under the microscope, exhibited no pus globules in its minute structure. On examining at the same time a portion of the mesentery, and carefully tracing up a branch of its mesenteric vein towards the intestine, this vein with its small branches was observed to be filled with shreds of coagulable lymph and semi-purulent matter. At the apex of both lungs, a few small cicatrices were found, as also some three or four small cretaceous masses. A few old and slight adhesions existed between the right lung and pleura costalis. The structure of the lungs and heart was perfectly healthy.

*Remarks upon the Report of the Post-mortem Examination.*—The statement of the appearances on the post-mortem examination, as drawn up by Dr. Isaacs, although correct in the main, is still deserving of a few remarks. In speaking of the liver, he does not state the peculiar appearance of the upper surface, which I think is worthy of note. On the convex surface, it presented a light opal shade—observable on both lobes—the lesser one being slightly *noduled,* but offering no resistance to the finger, upon being passed over it; the size of the liver was rather less than usual. He goes on to state "that it was then laid upon its inferior surface, and upon making an incision upon its upper or convex surface, *several small points of purulent matter were perceived.*" This statement certainly does not appear to me to give a complete view of the condition of things, inasmuch *as every branch of the portal vein in both lobes so cut, was literally gorged with purulent matter, as far as the eye could follow its ramifications.* Dr. Isaacs then further states, "that the trunk of the vena portæ was examined and presented the appearance and feel of a firm, hard cylinder, and when opened was found to be filled with coagulated lymph and semi-purulent matter, and a few small clots of blood. The vein on being traced into the right and left lobes of the liver, exhibited the same appearances in its branches, as far as their third and fourth divisions." This description of the appearances of this vein does not certainly accord with my impression derived at the time. The trunk of the portal vein was at its cut extremity partially supported by a cylindrical lining or false membrane of lymph, attached to the inner coat of the vein by shreds: this vein was imperfectly filled with purulent matter, much having run out. The cylindrical lining was found, when laid open, to extend throughout to its third and fourth divisions, the orifices were *filled* with *purulent* matter. I could not discern the *clots of blood* as discerned by Dr. Isaacs. Dr. Isaacs then makes mention subsequently (next day) of a small portion of the liver being placed under the microscope, "exhibiting no pus-globules in its minute structure," and "on examining at the same time a portion of the mesentery, and carefully tracing up a branch of its mesenteric vein towards the intestine, this vein with its small branches was observed to be filled with shreds of coagulated lymph, and semi-purulent matter." As I was not invited to these microscopic observations, I will here take the liberty of asking Dr. Isaacs how far the matter or pus globules were traced in the minute distribution of the portal vein in the liver? Does the *minute structure* of the liver, as above stated, include the minute distribution of the portal vein? or is the remark confined to other portions of that organ? I must infer that it has reference to the latter only, as these branches were gorged with purulent matter, as far as the eye could trace them. While upon microscopic inspections, I would beg leave to remark, that as no mention is made of the pus globules being of long standing, I presume it is conceded that the matter found in the post-mortem examination of the body of Dr. R., was of recent formation. That the matter as found in the depots in the mesentery was not of long standing is evident, both from the fact that he had enjoyed comparatively good health to the time of his late illness, except occasional bilious discharges from the bowels, as well as from the fact that the depots of matter above described were not encysted or supported by dense walls or distinct coverings; as they were barely sufficient to retain the accumulation, thereby showing that the pus as found in the depots was of rapid and recent formation.

Dr. Isaacs omitted, or probably it did not seem to him important, to detail the conversation I had with him at the time of conducting the post-mortem examination. I will, therefore, state, as it is important in confirming my views of the case, that at this stage of the dissection, after the removal of the stomach and intestines (for I had not before spoken to him in reference to the post-mortem), I requested him to remove the liver and its appendages carefully, and to place them in a basin for examination near the window, which he politely did. I then requested him after inspecting the convex surface of the liver, first to examine the capsule of Glisson, and the main ducts with the blowpipe, to ascertain their condition: they were sound. Secondly, I requested him to turn the liver over (convex side uppermost) and to make a deep incision transversely through the major lobe so as to divide the ramification of the branches of the portal vein, and to repeat the same in the lesser lobe; this also he did. They were found gorged with matter. Thirdly, I then requested him to pass a blowpipe into the main channel or vena portæ to ascertain if the branches so divided by the incision (and gorged with matter) were the ramifications of that vessel only; this was also done. Fourthly, I then asked him to make an incision diagonally to the first and longitudinally with the trunks of that vessel; this was done, and they also were found gorged with matter and cylindrical lymph. The same was repeated on the lesser lobes with similar results. Fifthly, I furthermore requested Dr. Isaacs to turn the liver over and examine the portal vein on its entrance into the liver, and to divide it quite up to its first divisions; they were, in like manner, found gorged with purulent matter, and contained the same cylindrical lymph formation.

